# Enhancing Thromboembolic Risk Prediction in Non-valvular Atrial Fibrillation: The Critical Role of Transesophageal Echocardiography (TEE)

**DOI:** 10.7759/cureus.76064

**Published:** 2024-12-20

**Authors:** Anas Hadari, Jaouad Nguadi, Hamid Jalal, Laila Bendriss

**Affiliations:** 1 Cardiology, Mohammed VI Military Hospital, Dakhla, MAR; 2 Cardiology, Mohammed V Military Teaching Hospital, Rabat, MAR; 3 Cardiology, Avicenna Military Hospital, Marrakesh, MAR

**Keywords:** aortic atheroma, cha2ds2-vasc score, nonvalvular atrial fibrillation, spontaneous echo contrast, the emptying velocity of the left atrial appendage, thromboembolic risk, transesophageal echocardiography (tee)

## Abstract

Introduction

Atrial fibrillation (AF), the most common cardiac arrhythmia, poses challenges in predicting thromboembolic risk. While the CHA_2_DS_2_-VASc (congestive heart failure, hypertension, age ≥ 75 years (doubled), type 2 diabetes mellitus, previous stroke, transient ischemic attack, or thromboembolism (doubled), vascular disease, age 65-74 years, and sex category) score remains essential, its limitations include failure to identify left atrial (LA) thrombus in some patients. Transesophageal echocardiography (TEE) provides superior detection of LA thrombi and thrombogenic factors compared to transthoracic echocardiography (TTE), improving risk stratification, especially in intermediate-risk groups. Our study highlights the value of TEE in addressing gaps left by clinical scoring systems in certain subgroups of patients.

Purpose and methodology

This descriptive, prospective study aims to evaluate the role of transthoracic and transesophageal echocardiography in stratifying thromboembolic risk in patients with non-valvular AF. A total of 100 patients, from two hospitals in Morocco, were included. Data were collected through clinical and paraclinical assessments, with echocardiography examining morphological and functional atrial parameters.

Results

Among the 100 patients, 73% were male, with a mean age of 67 years. AF was permanent in 84.8% of cases, with dyspnea and palpitations being the most common symptoms. Hypertension was the leading underlying cause. Echocardiographic findings showed a correlation between LA enlargement, reduced left atrial appendage (LAA) emptying velocities, and increased thromboembolic risk. In patients with low clinical scores, 30.7% exhibited echocardiographic signs of a thrombogenic environment, while protrusive aortic atheroma was more prevalent in those with higher clinical risk scores.

Discussion

The findings confirm the utility of echocardiography, particularly transesophageal, in detecting parameters associated with heightened thromboembolic risk, including LAA emptying velocities, spontaneous contrast, and aortic abnormalities. These echocardiographic markers, combined with clinical scores, may enhance the precision of risk stratification and allow for more targeted anticoagulation therapy.

Conclusion

Atrial fibrillation remains a common and potentially serious arrhythmia. Echocardiography provides valuable information that complements clinical risk stratification, especially for patients at moderate thromboembolic risk. This study highlights the benefit of incorporating echocardiographic parameters into risk assessment to optimize strategies for preventing thromboembolic events in patients with AF.

## Introduction

Atrial fibrillation (AF) is the most prevalent cardiac rhythm disorder, contributing to an elevated risk of cardiovascular morbidity and mortality, independent of underlying pathologies or associated risk factors [1].

While the clinical risk factors forming the basis of thromboembolic risk stratification (CHA_2_DS_2_-VASc (congestive heart failure, hypertension, age ≥ 75 years (doubled), type 2 diabetes mellitus, previous stroke, transient ischemic attack, or thromboembolism (doubled), vascular disease, age 65-74 years, and sex category) score) have been well-established and validated by the Atrial Fibrillation Investigators (AFI) and the Stroke Prevention in Atrial Fibrillation (SPAF) trials - and subsequently incorporated into the 2011 European and American guidelines - the role of echocardiography has started to gain increasing importance. This significance is highlighted in the 2020 updated AF guidelines, underscoring the need to better define its contributions and potentially integrate echocardiographic findings with clinical risk factors [2,3]. The ultimate goal is to more accurately identify patients who would benefit from long-term anticoagulation, thereby reducing the incidence of thromboembolic events [4].

Transesophageal echocardiography (TEE) is particularly valuable in detecting left atrial appendage (LAA) thrombus and assessing left atrial (LA) function, which are critical for stratifying thromboembolic risk and guiding anticoagulation decisions. Several studies have highlighted the incremental prognostic value of TEE findings when integrated with clinical risk scores, suggesting a more tailored approach to anticoagulation therapy. The integration of echocardiographic parameters into conventional risk stratification models could potentially lead to better identification of high-risk patients who would benefit from long-term anticoagulation, thereby reducing the incidence of thromboembolic events [5].

In light of these developments, the current study aims to evaluate the role of transthoracic and transesophageal echocardiography in stratifying thromboembolic risk in patients with non-valvular AF, with a focus on identifying specific echocardiographic parameters relevant to risk assessment.

## Materials and methods

Study design and setting

This descriptive, prospective study was conducted at the cardiology departments of the Avicenne Military Hospital in Marrakesh and the Mohammed VI Military Hospital in Dakhla, Morocco. The study focused on evaluating the role of transthoracic and transesophageal echocardiography in assessing thromboembolic risk among patients diagnosed with non-valvular AF.

Study population

A total of 100 patients with non-valvular AF were recruited for this study. The inclusion criteria were: (i) adults aged 18 years and older diagnosed with non-valvular AF; (ii) patients who provided informed consent to participate in the study; (iii) availability for scheduled follow-up consultations during the study period.

The exclusion criteria were: (i) patients with valvular heart disease, including rheumatic mitral stenosis and mechanical or bioprosthetic heart valves; (ii) individuals with a history of LA thrombus; (iii) patients currently undergoing anticoagulation therapy for conditions other than AF; (iv) pregnant women and individuals unable to give informed consent.

Data collection

Data were collected prospectively using a standardized operational sheet, capturing both clinical and paraclinical parameters, including echocardiographic findings, patient demographics, medical history, and associated risk factors. Follow-ups were conducted through scheduled consultations to monitor patient outcomes.

Ethical considerations 

The study protocol was reviewed and approved by the Cardiology Research Committee Institutional Review Board (IRB) with approval number 270592. The study adhered to the ethical guidelines for research involving human subjects. Written informed consent was obtained from all participants prior to inclusion in the study, ensuring confidentiality and voluntary participation.

## Results

This study included 100 patients, with a predominance of males (73 men and 27 women), resulting in a sex ratio of 2.7. The mean age of the population was 67 years. Patients over the age of 75 accounted for 18%, with the majority falling within the 65-74 age range (49%), and 33% of patients were younger than 64 years. AF was permanent in the majority of cases (84.8%). The primary symptoms reported were dyspnea and palpitations. Additionally, 15% of cases presented with AF secondary to cerebral embolic complications (stroke).

Hypertension was identified as the most common underlying cause of AF, accounting for nearly half of the cases, followed by dysthyroidism, diabetes, and chronic obstructive pulmonary disease (COPD). In 25% of cases, AF was classified as isolated, with no identifiable underlying cause.

Regarding thromboembolic risk, 43% of patients had a CHA_2_DS_2_-VASc score between 2 and 4, while 39% were classified as low risk, with a score between 0 and 1. Antithrombotic treatment, either vitamin K antagonists or direct oral anticoagulants (DOACs), was prescribed to 85% of the patients, whereas 15% did not receive anticoagulation therapy. For patients with permanent AF, a rate-control strategy was employed, with 84% requiring bradycardic medications, including digoxin, beta-blockers, or calcium channel blockers.

Echocardiographic data were analyzed using IBM SPSS Statistics (IBM Corp., Armonk, United States). Transthoracic echocardiography (TTE) revealed left ventricular systolic dysfunction in five patients and left ventricular hypertrophy in 24 patients (24% of cases). Morphological abnormalities of the left atrium were observed in most patients. Notably, an LA surface area ≥ 20 cm² and LA volume ≥ 35 ml/m² were significantly correlated with the clinical risk score, while the LA shortening fraction did not show a significant correlation with the CHA_2_DS_2_-VASc score.

Among patients with a clinical score < 2, 30.7% exhibited echocardiographic signs of a thrombogenic environment (spontaneous contrast and reduced left intra-atrial velocities). Conversely, 18.7% of patients with a score ≥ 2 did not present any thrombogenic features. A correlation was established between various echocardiographic parameters and the level of clinical risk (Table 1).

**Table 1:** Echocardiographic abnormalities correlated with the CHA2DS2-VASc clinical score LA: Left atrium; LAA: left atrial appendage; NS: Not significant

CHA_2_DS_2_-VASc score	0-1 (n=39)	2-4 (n=43)	5-9 (n=18)	P-value
					Indexed LA diameter ≥ 2.5 cm/m²	14 (35.8%)	37 (86.0%)	15 (83.3%)	0.04
Indexed LA shortening fraction ≤ 25%	29 (74 %)	37 (86.0%)	12 (66.6%)	NS
LA surface area ≥ 20 cm²	20 (51.2%)	23 (53.4%)	18 (100%)	0.01
Indexed LA volume ≥ 35 ml/m²	24 (61.5%)	26 (60.4%)	10 (55.5%)	0.03
LAA surface area > 5 cm²	0	11 (25.5%)	10 (55.5%)	0.04
Intra-LAA velocities < 25 cm/s	12 (30.7%)	35 (81.3%)	15 (83.3%)	0.01
Spontaneous contrast present	4 (10.2%)	35 (81.3%)	12 (66.6%)	0.02
Aortic atheroma ≥ 4 mm	0	23 (53.4%)	12 (66.6%)	0.004

TEE revealed that patients with low clinical scores (0-1) generally had normal-sized left atria and an absence of aortic atheroma. The presence of protrusive aortic atheroma was a significant discriminating factor, correlating strongly with higher clinical scores. Spontaneous contrast was more frequently observed in patients with a clinical score ≥ 2 (p=0.001) but was not directly correlated with score severity. However, the density of spontaneous contrast showed a strong correlation with clinical score severity (p=0.003) (Table 2). The emptying velocity of the LAA was inversely proportional to clinical score severity, with a significant correlation. This velocity was also correlated with the presence and severity of spontaneous contrast (p=0.001). No LAA thrombus was detected, likely due to the majority of patients receiving anticoagulation therapy at the time of inclusion.

**Table 2:** Grade of spontaneous contrast according to the clinical score

CHA_2_DS_2_-VASc score	0-1 (n=39)	2-4 (n=43)	5-9 (n=18)
Contrast grades			
Grade 0	2	2	0
Grade 1	4	5	0
Grade 2	0	4	1
Grade 3	0	3	3

In summary, 40 patients (40%) were identified as having a high thromboembolic risk based on echocardiographic findings (presence of thrombus and/or dense spontaneous contrast, dampened emptying velocity < 20 cm/s, or protrusive aortic atheroma). These patients were distributed as follows: 18 patients with a clinical score ≥ 5, representing all patients in this category, and 22 patients with a score between 2 and 4, accounting for nearly half of this group (Table 3).

**Table 3:** Correlation between the clinical score and the presence of high thromboembolic risk echocardiographic factors

CHA_2_DS_2_-VASc score	Patients at high thromboembolic risk in echocardiography (n=40)
0-1	4 (10.2%)
2-4	22 (51.1%)
≥ 5	18 (100%)

The prevalence of morphological abnormalities in this study was 21% for LAA dilation and 35% for protrusive aortic atheroma. Among the low clinical score group (0-1), 30.7% exhibited these abnormalities. There was a strong correlation between the clinical score and echocardiographic findings when the clinical score was ≥ 2 (p=0.001). The key discriminative factors in this study were the density of spontaneous contrast and the presence of protrusive aortic atheroma.

Anticoagulant therapy was initiated in four patients with a clinical score of 0 due to reduced LA emptying velocity and grade 2 spontaneous contrast. During follow-ups at one, three, and six months, as well as at one year, no embolic events were recorded, and no patients were lost to follow-up.

## Discussion

AF is associated with a significant risk of thromboembolic events, which necessitates the initiation of anticoagulant therapy to mitigate the risk. However, anticoagulation therapy carries its own risks, particularly due to the narrow therapeutic window, which can lead to complications if not carefully managed. As a result, current guidelines from major cardiology societies, including the American College of Cardiology (ACC), the American Heart Association (AHA), the European Society of Cardiology (ESC), and the European Heart Rhythm Association (EHRA), recommend anticoagulants only for patients with established thromboembolic risk factors [1]. The CHA_2_DS_2_-VASc score, a widely accepted clinical risk stratification tool, plays a critical role in identifying patients at higher risk for stroke and thromboembolic events and is recommended by these guidelines [2].

The CHA_2_DS_2_-VASc score, developed from studies such as those by the AFI and the SPAF, has become the most validated and commonly used model for stroke risk stratification in AF patients [3]. However, its utility in predicting non-cerebral embolism has not been thoroughly studied, and the understanding of clinical and echocardiographic features that increase the risk of peripheral embolism remains limited [4].

In the SPAF I study, which followed 568 patients with non-valvular AF for an average of 1.3 years, LA dilation (≥ 2.5 cm/m² on time-motion (TM)) and reduced systolic function, measured by the left ventricular shortening fraction (a fraction < 25%), were identified as important risk factors for thromboembolic events [5]. Our study reinforces the importance of LA dilation in thromboembolic risk assessment, with LA area and volume emerging as significant parameters. However, we did not observe a strong correlation with the shortening fraction, likely due to the smaller sample size in our study.

TEE is an invaluable tool for assessing atrial structure and thromboembolic risk, particularly for visualizing the LAA. Data from the SPAF III trial demonstrated that patients with LA velocities of less than 20 cm/s had a 75% likelihood of spontaneous contrast within the LA, highlighting the utility of TEE in stratifying thromboembolic risk [6]. Similarly, Mügge's study reported that LAA velocities below 25 cm/s were associated with spontaneous contrast in 80% of cases [7]. Fatkin’s work further solidified this connection, showing that severe spontaneous contrast (grade 4) or the presence of a thrombus in the LAA was correlated with velocities under 20 cm/s [8]. Our findings are consistent with these observations, demonstrating a significant correlation between LAA emptying velocities and the presence of spontaneous contrast, particularly in cases of dense contrast.

Another critical contribution of TEE is the evaluation of the thoracic aorta for the presence of aortic atheroma, which is common in elderly AF populations, with prevalence rates ranging from 46% to 57% [9-11]. Aortic atheroma significantly increases the risk of embolic events, particularly in cases where the plaques are protrusive (≥ 4 mm) or complex (involving thrombus or ulceration), with studies showing that these lesions can raise the risk of embolism by three to nine times in patients over 60 years old [1,12-14]. Blackshear's study found that patients with both LAA abnormalities and protrusive aortic atheroma had a significantly higher annual risk of vascular events (approximately 20%) compared to those with spontaneous contrast alone (7.5%) or protrusive atheroma alone (12%) [15]. Zabalgoitia's research similarly demonstrated that the prevalence of LAA and aortic anomalies is low in patients with low clinical risk (15-20%) but increases to 45% and 40%, respectively, in those with high clinical risk [6]. Our study also identified a 39% prevalence of protrusive aortic atheroma in patients with moderate to high clinical risk, which aligns with these findings.

A study conducted in the United States compared two groups of AF patients: those who experienced non-cerebral embolic events and those who had strokes. The CHA_2_DS_2_-VASc score was similar in both groups (2.5±1.1 vs. 2.3±1.3), with 20% of patients in each group having scores below 1, and only 5% scoring above 4 [16]. However, patients in the non-cerebral embolism group had higher rates of certain clinical characteristics, such as heart failure, age over 75, and a history of hypertension, compared to the stroke group. Additionally, echocardiographic features such as LAA dilation, reduced emptying velocities, spontaneous contrast, and aortic atheroma were more prevalent in the non-cerebral embolism group.

An American-Chinese study involving AF patients who underwent TEE before cardioversion or catheter ablation identified two groups: those with and those without LAA thrombus. The presence of thrombus was associated with older age, LAA dilation, spontaneous contrast, and lower LAA emptying velocities (with a cut-off of 21.5 cm/s) [17]. These findings also support our results, where we observed spontaneous contrast in 36% of patients with a CHA_2_DS_2_-VASc score of 0, although no thrombus was detected.

By comparing the clinical and echocardiographic scores, it becomes evident that there is no strong correlation between the CHA_2_DS_2_-VASc score and the presence of spontaneous contrast or thrombus in the LAA. For instance, in cases where the CHA_2_DS_2_-VASc score is 0, the incidence of atrial thrombus remains low at around 3%, while the rate of spontaneous contrast is higher at 24% [17]. In our study, among patients with a CHA_2_DS_2_-VASc score of 0, we found that 10.2% exhibited grade 1 or 2 spontaneous contrast, but none had evidence of an atrial thrombus.

Although the CHA_2_DS_2_-VASc score is widely used to predict thromboembolic risk in AF patients, its ability to specifically predict atrial thrombus formation remains controversial. Some studies suggest that the score underestimates the likelihood of thrombus formation [16,18,19], while others present conflicting findings. However, it is important to emphasize that while the CHA_2_DS_2_-VASc score is a reliable indicator of overall thromboembolic risk, it may not be the most accurate tool for predicting thrombus formation specifically.

This study has several limitations that should be acknowledged. Firstly, TEE is highly operator-dependent, meaning that the accuracy and quality of the imaging can vary significantly depending on the experience and skill of the operator. This variability may introduce potential biases in the assessment of thromboembolic risk based on echocardiographic parameters. Additionally, TEE is known to be volume-dependent, as the LAA visualization can be affected by the patient's hemodynamic status at the time of the examination. These factors could impact the reproducibility of our findings.

Furthermore, the degree of anticoagulation in patients at the time of the TEE study could influence the detection of thrombus or spontaneous echo contrast. Variability in anticoagulation management, such as differences in the intensity and duration of anticoagulant therapy prior to imaging, may have affected the echocardiographic evaluation of thromboembolic risk. Future studies with standardized anticoagulation protocols are needed to minimize this confounding factor.

In conclusion, while the CHA_2_DS_2_-VASc score is indispensable for clinical risk stratification, echocardiographic findings, particularly from TEE, offer additional insights into thromboembolic risk, particularly in detecting spontaneous contrast, reduced LAA emptying velocities, and aortic atheroma. These echocardiographic markers, when combined with clinical data, can help refine anticoagulation strategies and reduce thromboembolic events in AF patients.

## Conclusions

AF remains the most prevalent rhythm disorder, significantly contributing to increased cardiovascular morbidity and mortality. Both TTE and, more critically, TEE are indispensable tools in clinical practice. These imaging modalities offer enhanced visualization of atrial morphology and function, enabling more accurate assessment and stratification of thromboembolic risk. This precision supports more informed decision-making regarding anticoagulation therapy and improves overall management strategies for patients with AF.
